# Predictive Value of Serum Neurofilament Light Chain Levels in Anti-NMDA Receptor Encephalitis

**DOI:** 10.1212/WNL.0000000000207221

**Published:** 2023-05-23

**Authors:** Juliette Brenner, Sara Mariotto, Anna E.M. Bastiaansen, Manuela Paunovic, Sergio Ferrari, Daniela Alberti, Marienke A.A.M. de Bruijn, Yvette S. Crijnen, Marco W.J. Schreurs, Rinze F. Neuteboom, Jan G.M.C. Damoiseaux, Juna M. de Vries, Maarten J. Titulaer

**Affiliations:** From the Department of Neurology (J.B., A.E.M.B., M.P., M.A.A.M.d.B., Y.S.C., R.F.N., J.M.d.V., M.J.T.), Erasmus University Medical Center, Rotterdam, the Netherlands; Neurology Unit (S.M., S.F., D.A.), Department of Neurosciences, Biomedicine and Movement Sciences, University of Verona, Italy; Department of Immunology (M.W.J.S.), Erasmus University Medical Center, Rotterdam; and Central Diagnostic Laboratory (J.G.M.C.D.), Maastricht UMC+, the Netherlands.

## Abstract

**Background and Objectives:**

Determinants of disease activity and prognosis are limited in anti-NMDA receptor (NMDAR) encephalitis. Neurofilament light chains (NfL) are markers of axonal damage and have been identified as valuable biomarkers for neurodegenerative and other neuroinflammatory disorders. We aimed to investigate serum NfL levels in patients with anti-NMDAR encephalitis as a biomarker for disease severity and outcome.

**Methods:**

In this retrospective study, NfL values were measured in all available pretreatment serum and paired CSF samples of the nationwide anti-NMDAR encephalitis cohort. The values were analyzed in duplicate using single-molecule array and compared with measurements in healthy references. Follow-up sera were tested to analyze longitudinal responsiveness, if at least available from 2 time points after diagnosis. Serum NfL levels were compared with data on disease activity (seizures, MRI, and CSF findings), severity (modified Rankin Scale [mRS] score, admission days, and intensive care unit admission), and outcome (mRS score and relapses), using regression analysis.

**Results:**

We have included 71 patients (75% female; mean age 31.4 years, range 0–85 years) of whom pretreatment serum samples were analyzed. Paired CSF samples were available of 33 patients, follow-up serum samples of 20 patients. Serum NfL levels at diagnosis were higher in patients (mean 19.5 pg/mL, 95% CI 13.7–27.7) than in references (mean 6.4 pg/mL, 95% CI 5.8–7.2, *p* < 0.0001). We observed a good correlation between serum and CSF NfL values (*R* = 0.84, *p* < 0.0001). Serum NfL levels and age correlated in patients (Pearson *R* = 0.57, *p* < 0.0001) and references (*R* = 0.62, *p* < 0.0001). Increased NfL values were detected in patients post–herpes simplex virus 1 encephalitis (mean 248.8 vs 14.1 pg/mL, *p* < 0.0001) and in patients with brain MRI lesions (mean 27.3 vs 11.1 pg/mL, *p* = 0.019). NfL levels did relate to the long-term follow-up (mRS score at 12 months; β_NfL_ = 0.55, *p* = 0.013), although largely explained by the effect of age on NfL levels and prognosis. In serial samples, NfL values did roughly follow clinical disease activity, albeit with delay.

**Discussion:**

Increased serum NfL levels reflect neuroaxonal damage in anti-NMDAR encephalitis. No relationship was identified with disease severity, whereas the association with outcome was confounded by age. The implied role of sampling timing on NfL levels also limits the applicability of NfL as a prognostic marker.

Anti-NMDA receptor (NMDAR) encephalitis is a complex immune-mediated disorder characterized by antibodies in the CSF against the ionotropic glutamate receptor type 1 subunit of the NMDAR. Clinical features include behavioral changes, cognitive impairment, seizures, language disorders, movement disorders, and autonomic dysfunctions. Anti-NMDAR encephalitis can occur as a paraneoplastic phenomenon (most often associated with ovarian teratomas), postinfectious after herpes simplex virus (HSV) encephalitis or sporadically.^1^ The disease is treatable by removing the trigger (if paraneoplastic) and administering immunotherapy. Still, patients might require admission to the intensive care unit (ICU) during the acute stage. Many patients experience persisting neurologic deficits, and 12% of cases relapse within 2 years.^2^ The outcome of anti-NMDAR encephalitis has previously been related to clinical factors like the requirement of ICU admission, treatment delay, and a lack of response to first-line immunotherapy.^2,3^ CSF leukocyte count and antibody titers correlate with outcome and clinical relapses.^3,4^ However, titers do not consistently reflect disease activity.^5^ Treatment decisions are currently based on clinical assessment since, despite several attempts, biomarkers for disease severity and prognosis are very limited.^6^

Neurofilaments, and in particular the light chain subunit, are released from axons after acute damage. Neurofilament light chain (NfL) levels have therewith been identified as a useful biomarker for disease activity and prognosis in different neuroinflammatory and degenerative neurologic disorders.^7^ The strong correlation between CSF and serum NfL values and the high sensitivity of novel diagnostic techniques, allowing to quantify the lower levels detectable in serum, seem to expand the applicability of serum NfL as a biomarker.^8^ The preanalytical stability of NfL values (i.e., to delayed freezing and repeated thawing/freezing cycles) additionally raises the potential to investigate NfL as a biomarker.^9^ In this study, we investigate serum NfL levels at diagnosis and follow-up in patients with anti-NMDAR encephalitis to evaluate whether this biomarker of ongoing axonal damage correlates with disease severity and long-term outcome.

## Methods

### Study Participants and Sample Selection

As the national referral center for autoimmune encephalitis of the Netherlands, accredited as the European Reference Network site (European Reference Network for Rare Immunodeficiency, Autoinflammatory and Autoimmune Diseases Network), we take note of all nationwide diagnoses of anti-NMDAR encephalitis. We have targeted all Dutch patients complying with the criteria for definite anti-NMDAR encephalitis,^10^ based on (1) the availability of a sufficient amount of serum from the time of diagnosis, (2) serum drawn before the start of immunotherapy, and (3) relevant clinical data of at least 4 months after diagnosis (eFigure 1, links.lww.com/WNL/C731). All eligible patients had previously consented to be in the nationwide anti-NMDAR encephalitis cohort and have been phenotyped clinically well (eTable 1).^11^ We compared the data with a healthy reference group (n = 61; 70% female; mean age 41.9 years, range 25–67 years) and with previously suggested age-based cutoff values.^12–14^ To correlate serum with CSF, we tested all available pretreatment CSF samples drawn within 48 hours from the serum sample. To investigate NfL longitudinally, we selected those patients of whom we had sufficient amounts of sera from at least 2 different time points after diagnosis.

### Clinical Parameters

Extensive clinical data had been collected as part of our nationwide study.^11^ Age at onset, preceding HSV encephalitis, concomitant tumors, the presence of seizures or movement disorders, cerebral MRI abnormalities, and antibody titers were considered potentially relevant covariates for NfL levels. Maximum modified Rankin Scale (mRS) scores, duration of hospital admission, and the need for ICU admission were used as measures for disease severity. Short- and long-term outcomes were quantified as the mRS score at 4 and 12 months after diagnosis, respectively. A relapse was defined as the (re)emergence or worsening of clinical symptoms fitting the diagnostic criteria for anti-NMDAR encephalitis, after a period of at least 2 months of improvement or stabilization, combined with the confirmation of anti-NMDAR antibodies in CSF.^2,11^

### Procedures for NMDAR Antibody and NfL Measurements

Anti-NMDAR antibodies were detected using cell-based assays (Euroimmun, AG, Lübeck, Germany) in CSF, and confirmed by immunohistochemistry, as described before.^11^ All patients had antibodies in CSF. NfL concentration in serum and CSF was measured in duplicate using single-molecule array NfL-light kit with SR-X immunoassay analyzer (Quanterix Corp., Billerica, MA), as previously described,^15^ by investigators blinded to clinical data. A Comparison was made with sera from 61 healthy controls. The mean intra-assay coefficient of variation (CoV) of duplicates and interassay CoV were 6.7% and 6.4%, respectively. Samples with CoV above 20% were reanalyzed.

### Standard Protocol Approvals, Registrations, and Patient Consents

This retrospective study was waived and declared non–complicit to the Medical Research Involving Humans Subjects Act by the Institutional Review Board of Erasmus MC. Written informed consent was obtained from all patients.

### Statistics

The data on NfL values in serum and CSF were logarithmically transformed to adjust for skewness of the distribution. The descriptive statistics provided in this paper are centered around the geometric means. The correlation between NfL levels in serum and CSF was investigated by calculating Pearson correlation coefficient. A good correlation allowed serum NfL to be used as a surrogate biomarker. The serum NfL levels of the patients were compared with healthy adult references, as well as with age-based cutoff values from the literature, also including pediatric references.^12–14^ The known influence of age on NfL levels was confirmed by fitting a linear regression model. The rest of the analyses were corrected for this effect by the addition of age as a covariate. As the less extensively investigated effect of age on NfL in children does not seem strictly linear in the lowest age range, and the included healthy references were adults, we also performed all analyses in the subgroup of adult patients.

The relationship between the independent variables tumor, preceding HSV1 infections, and visible MRI abnormalities and the dependent variable serum NfL and the relationship between serum NfL levels (independent variable) and duration of hospital admission were tested with variants of linear regression models, univariable and multivariable with age as a covariate. Because of the reported effect of an HSV1 encephalitis on both NfL levels and prognosis of anti-NMDAR encephalitis,^16,17^ we have left these patients out of the analyses to determine the prognostic value of serum NfL in anti-NMDAR encephalitis (eFigure 1, links.lww.com/WNL/C731). Logistic regression analysis was applied to investigate the relationship between serum NfL at diagnosis and the need for ICU admission, as measures of disease severity. The predictive value of early NfL levels for maximum disease severity (maximum mRS score), outcome (mRS score at 4 and 12 months after disease onset), and time to recovery (improving to an mRS score ≤2) was explored with ordinal regression analysis. Patients with an mRS score >2 before disease onset were excluded from the latter analyses as we would not be able to determine the outcome specifically related to the anti-NMDAR encephalitis (eFigure 1).

### Data Availability

Any data not published within this article are available at the Erasmus University Medical Center. Patient-related data will be shared on reasonable request from any qualified investigator, maintaining anonymity of the individual patients.

## Results

We included 71 patients with anti-NMDAR encephalitis (75% female; mean age 31.4 years, range 0–85 years; Table 1), representative of the complete national cohort (eTable 1, links.lww.com/WNL/C731).

**Table 1:**
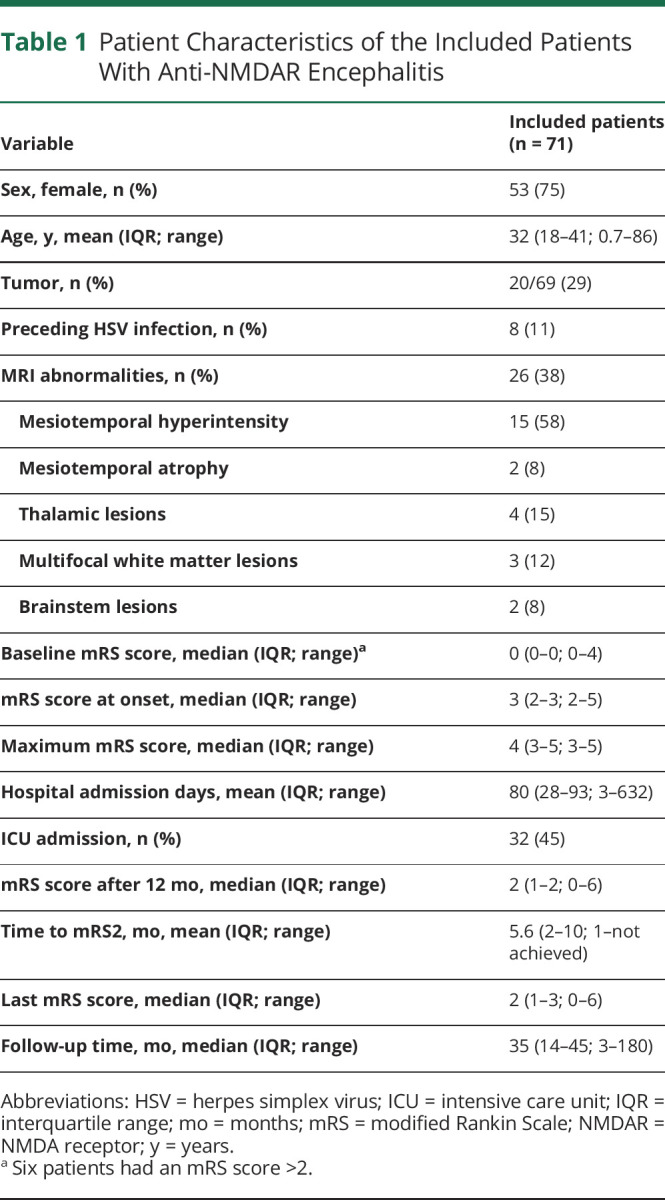
Patient Characteristics of the Included Patients With Anti-NMDAR Encephalitis

### NfL Levels and Associated Clinical Factors

**Figure 1:**
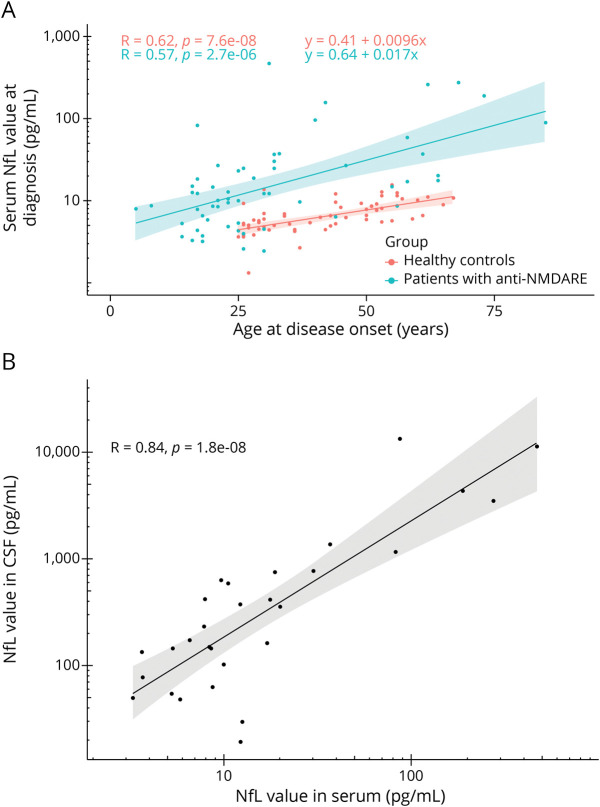
Serum NfL Correlation With Age and CSF NfL levels in serum correlate positively with age (A) and CSF (B). NfL = neurofilament light chain; NMDARE = NMDA receptor encephalitis.

**Figure 2:**
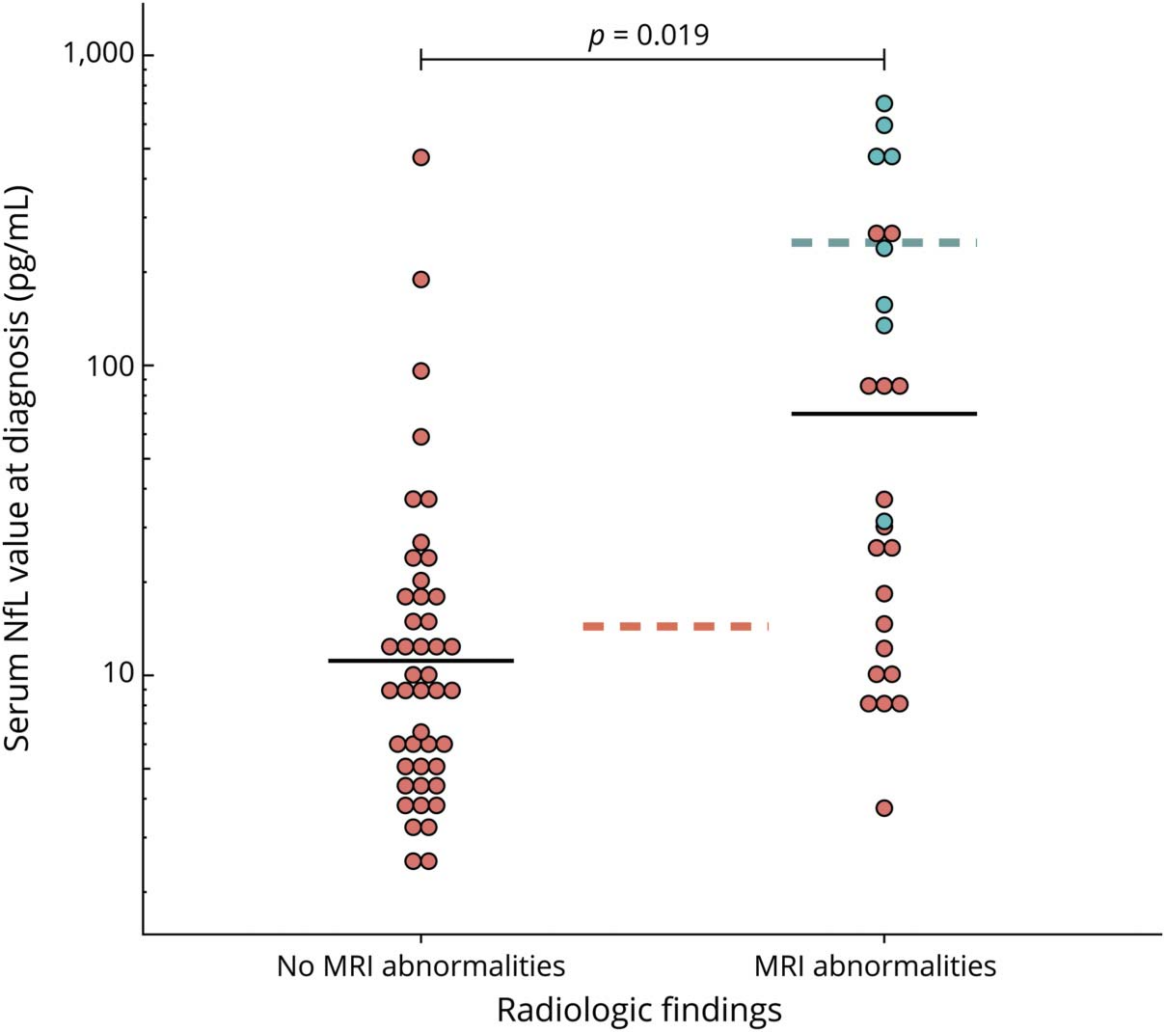
Serum NfL Related to Radiologic Findings Patients with anti-NMDAR encephalitis with MRI abnormalities had higher NfL levels in serum (*p* = 0.019; geographic means of patients with and without MRI abnormalities are represented by the black horizontal lines). Patients with a preceding HSV1 encephalitis (depicted in blue; all with MRI abnormalities) had even higher NfL levels in serum compared with patients without preceding a preceding HSV1 encephalitis (*p* < 0.0001; the geographic means of patients with and without a preceding HSV1 encephalitis are represented by the blue and red dotted horizontal lines, respectively). HSV = herpes simplex virus; NfL = neurofilament light chain; NMDAR = NMDA receptor.

The serum NfL concentration at diagnosis was higher in patients with anti-NMDAR encephalitis (mean 19.5 pg/mL, 95% CI 13.7–27.7) than in healthy controls (mean 6.4 pg/mL, 95% CI 5.8–7.2, *p* < 0.0001). Serum NfL values increased with increasing age at sampling in both patients (Pearson *R* = 0.57, *p* < 0.0001) and healthy controls (*R* = 0.62, *p* < 0.0001; Figure 1A). Serum and CSF NfL levels (n = 33) showed a good correlation (Pearson *R* = 0.84, *p* < 0.0001; Figure 1B). Patients with post-HSV1 anti-NMDAR encephalitis had higher serum NfL values than those without a preceding infection (mean 248.8 vs 14.1 pg/mL, *p* < 0.0001; Figure 2). Serum NfL levels were significantly higher in patients with cerebral MRI lesions compared with patients without (mean 27.3 vs 11.1 pg/mL, *p* = 0.019, patients with post-HSV1 encephalitis were not included in this analysis; Figure 2). These effects were similar when age was added to the analysis as a covariable (β_HSV_ = 2.7, *p* < 0.0001, β_MRI_ = 0.70, *p* = 0.012; Table 2). Analyzing these results in a slightly different way, using dichotomous age-based cutoff values, confirmed these results: patients with increased serum NfL levels (n = 39 [55%]) more frequently had a preceding HSV1 encephalitis (21% vs 0%, *p* = 0.019) and more frequently had MRI abnormalities (54% vs 16%, *p* = 0.002), compared with patients with serum NfL levels below the cutoff (eTable 2, links.lww.com/WNL/C731).

**Table 2:**
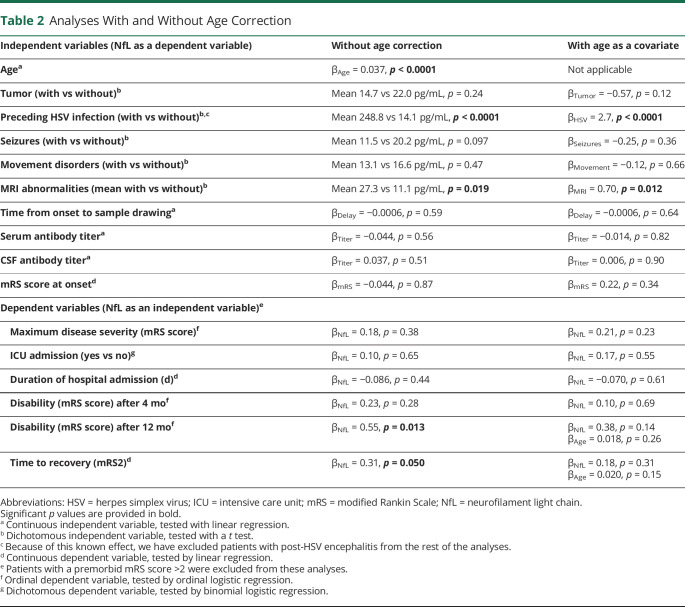
Analyses With and Without Age Correction

The presence of concomitant tumors, seizures, and movement disorders, the delay between symptom onset and sample drawing, and serum and CSF antibody titers did not significantly relate to NfL levels, with or without age as covariable (Table 2 and eFigures 2–4, links.lww.com/WNL/C731). A subgroup analysis of only the adult patients (n = 59), to account for different behavior of NfL as serum biomarker in children, did not provide different results (eTable 3, links.lww.com/WNL/C731).

### The Prognostic Value of NfL for Disease Severity and Outcome

NfL levels at diagnosis did not associate with markers for disease severity: it did not significantly differ between patients who needed ICU admission or not and did not relate to the maximum mRS score over the course of the disease (eFigure 5, links.lww.com/WNL/C731) nor the duration of hospital admission (eFigure 6). Similarly, no relation was noted between NfL levels at diagnosis and disability (mRS score) 4 months after disease onset (eFigure 7).

**Figure 3:**
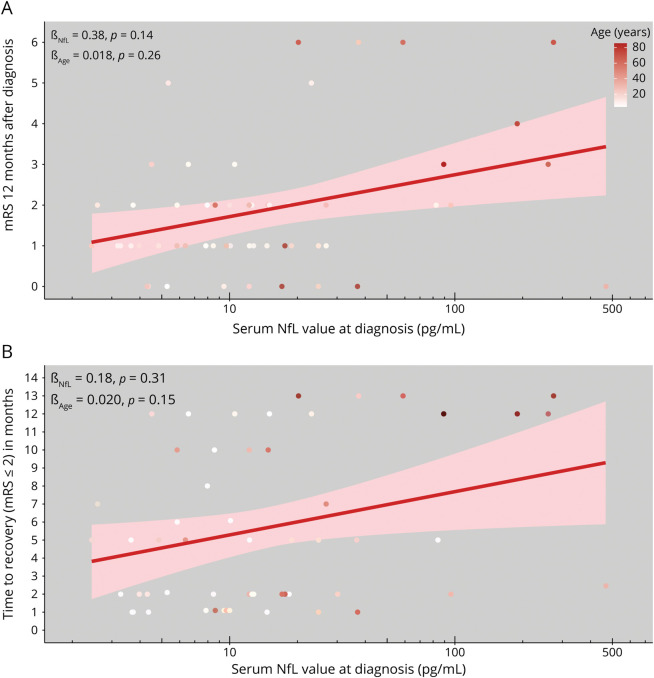
NfL, Age, and Long-term Outcome Higher NfL levels in serum were correlated with a worse outcome (higher mRS score) after 12 months (A) and a longer time to recovery (B). As can be seen by the colored dots, this was largely influenced by the age at onset. Correction for age at onset negated the significant association. mRS = modified Rankin Scale; NfL = neurofilament light chain.

In univariable analysis, NfL serum levels at diagnosis were related to the outcome after 12 months (β_NfL_ = 0.55, *p* = 0.013) and the time until recovery (to an mRS score ≤2; β_NfL_ = 0.31, *p* = 0.050), although this seemed largely attributed to the effect of age at disease onset (β_NfL_ = 0.38, *p* = 0.14 and β_Age_ = 0.018, *p* = 0.26 for outcome after 12 months, Figure 3A; β_NfL_ = 0.18, *p* = 0.31 and β_Age_ = 0.020, *p* = 0.15 for recovery time, Figure 3B; Table 2). These findings were confirmed when applying dichotomous age-based cutoff values (*p* = 0.069 for outcome after 12 months, *p* = 0.14 for recovery time; eTable 2, links.lww.com/WNL/C731), and a subgroup analysis of the adult patients showed no different results either (eTable 3).

### NfL in Longitudinal Follow-up Sera

**Figure 4:**
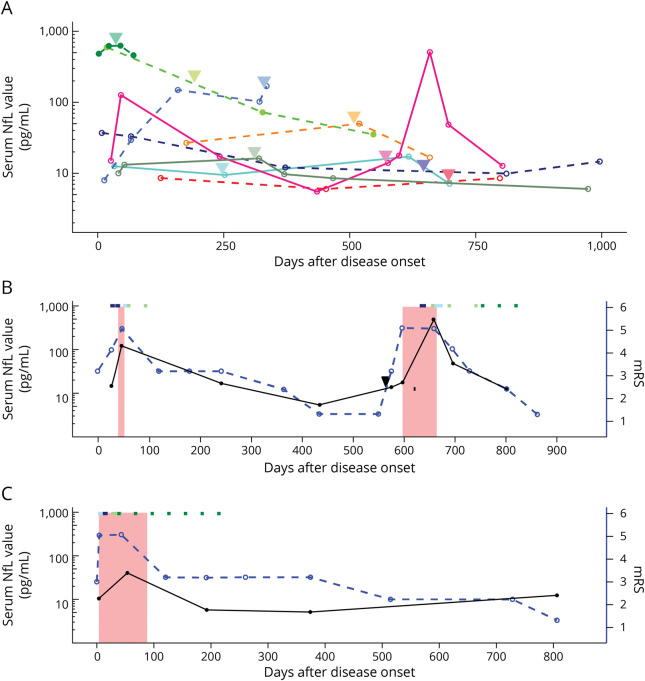
Longitudinal NfL Levels in Serum In all patients with a relapse (A), marked by the arrowheads. In 2 exemplary patients (B and C), we see an increase in NfL while admitted to the ICU (ICU admission annotated in red). The increase measured at the moment of relapse in patient B is similar to the one in the still-improving patient (C), without a relapse. The considerable increase is only seen later during the relapse. The treatment regime is represented by the colored squares at the top of the figure; IV methylprednisolone courses in light blue, immunoglobulins in dark blue, rituximab in light green, and cyclophosphamide in dark green. ICU = intensive care unit; mRS = modified Rankin Scale; NfL = neurofilament light chain.

**Figure 5:**
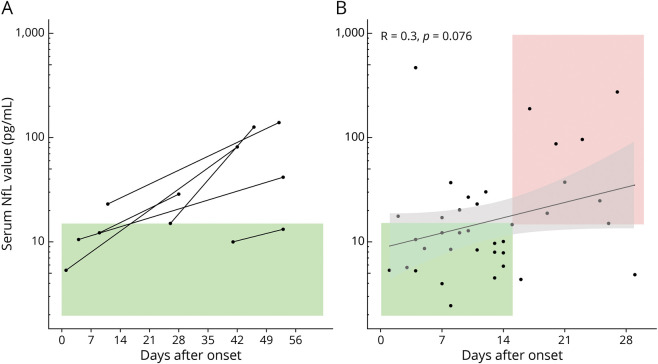
Details on Timing of NfL Measurements In all patients with multiple serum samples in the first 2 months after diagnosis, we see that the second measurements, starting at 28 days after diagnosis, exceed the normal range (A). The majority of all samples taken within the first 2 weeks after onset fall in the range of the healthy references (annotated with the green square; B). NfL = neurofilament light chain.

We included a total of 58 follow-up samples of 20 patients, of whom 10 had had at least 1 relapse of encephalitis (Figure 4A), and 10 had a monophasic course. When monitoring NfL levels over time, we noted that NfL values often increased considerably in the weeks after onset, especially while on the ICU, and had a subsequent decrease over time, more pronounced in patients discharged from the ICU (Figure 4, B and C, eFigures 8 and 9, links.lww.com/WNL/C731). Of interest, in an illustrative patient with a relapse, the main increase of NfL was seen only after the onset of symptoms (both in the initial episode and at relapse; Figure 4B). The suggestion of increase at the moment of onset of the relapse was similar to another patient who did not experience a relapse (Figure 4C). When focusing on the repeated serum measurements within the first months after disease onset, we see an increase of NfL levels up to 4–6 weeks (Figure 5A). This is in line with the observation that the majority of serum NfL measurements within the first weeks fall within the range of the healthy references, as opposed to the measurements after 2–4 weeks (Figure 5B).

## Discussion

In this study, we have investigated serum NfL as a biomarker in a large cohort of well-characterized patients with anti-NMDAR encephalitis. We demonstrate several important aspects: (1) although serum NfL levels are increased in patients with anti-NMDAR encephalitis, these do not provide independent prognostic value at diagnosis, neither for maximum severity nor for long-term outcome, and (2) serum NfL can be used to monitor the activity of disease in the chronic phase. However, the timing of serum NfL sampling has an influence on the values found, complicating the use as a biomarker to identify relapses early.

We have first established that serum NfL levels are increased in patients with anti-NMDAR encephalitis compared with the general population. Identified associations between NfL levels and age, a preceding HSV1 encephalitis, and radiologic signs of tissue damage are all in line with what we would expect, NfL being a marker of tissue injury associated with neuroaxonal damage.^8,17^

We identified no association between NfL levels at diagnosis and measures of maximum disease severity. In serial samples of patients admitted to the ICU, NfL levels increased within the first weeks; however, the initial values at diagnosis had no predictive value for ICU admission. Using univariable analysis, an association between serum NfL values and outcome after a year seemed to be present. As we and others have identified age as a factor associated both with higher NfL levels and with longer time to recovery, correction for age at onset was warranted.^11^ This explained at least the larger part of the difference in NfL levels, and no independent relationship between NfL and outcome at 12 months was identified.

These findings correspond partly with the literature. Whereas other studies also negate the association between initial NfL levels, albeit in CSF, and disease severity,^15,18^ 2 studies do associate NfL levels with disease severity (i.e., ICU admission).^19,20^ The referred samples in one were of the moment of determining severity and did not precede or predict disease severity (i.e., at diagnosis).^19^ Two of the mentioned studies, in homogeneous cohorts of patients with anti-NMDAR encephalitis, also described no applicability of NfL levels in CSF or serum as a biomarker for outcome.^18,20^ Two other studies found a correlation between NfL levels in diagnostic CSF samples and long-term outcome, even after (partial) correction for age, albeit in heterogeneous cohorts of patients with autoimmune encephalitis or paraneoplastic syndromes with diverse pathophysiologic mechanisms (not limited to anti-NMDAR encephalitis).^21,22^

The observed NfL increase in the weeks after symptom onset was previously observed in a cohort of patients with anti-NMDAR encephalitis.^19^ This might suggest that axonal damage is not a hyperacute initial feature of the disease causing clinical symptoms; rather, serum NfL levels likely reflect an integral measure of antecedent and ongoing neuronal damage. This additionally discourages the deployment of NfL as a biomarker, as the timing of sampling largely affects the values found. Although the longitudinal data are limited, we provide some data to suggest that the same delay in increase hampers the use of serum NfL as a marker to predict relapses. As serum levels do often increase, a delayed NfL measurement may be used as a marker to differentiate between a relapse, pseudorelapse (i.e., due to infection), or persisting neurologic symptoms. As serum NMDAR antibodies are not very reliable,^4^ and CSF NMDAR antibody titers at remission are often not available, this could still be very valuable to decide on escalation of treatment or installment of maintenance immunotherapy.

Our study has limitations, mainly related to the sample size and retrospective design. Although we have included all available pretreatment samples of our nationwide cohort, anti-NMDAR encephalitis is a rare disease, and the consequentially moderate sample size limits the power of our analyses. The retrospective study design did not allow us to monitor NfL values at regulated time points, and the longitudinal analysis is based on a limited subgroup only. In addition, follow-up was relatively short, and we did not perform regular imaging at consistent intervals, so we were unable to correlate NfL levels with lesion load and brain volume loss over time. Last, we used the mRS to quantify disability and outcome, which, despite being the most commonly used scale, is crude and not specific for this condition. More sensitive (cognitive) measures might yield different results correlating NfL values and disability. Prospective, structured follow-up could solve the majority of these limitations in the future.

In conclusion, axonal damage is a feature of active anti-NMDAR encephalitis, and measuring serum NfL might prove helpful in clinical practice to identify active disease and monitor recovery. NfL levels are no independent predictors for disease severity or outcome. As the timing of sampling seems to have a large effect on NfL values, the use of single values in prediction of disease severity, outcome, or relapses is complicated.

## Glossary

CoV: coefficient of variation
HSV: herpes simplex virus
ICU: intensive care unit
mRS: modified Rankin Scale
NfL: neurofilament light chain
NMDAR: NMDA receptor
